# Haloperidol-Induced Catalepsy and Its Correlations with Acetylcholinesterase Activity in Different Brain Structures of Mice

**DOI:** 10.3390/neurolint16060125

**Published:** 2024-12-05

**Authors:** Brenda Rufino da Silva, Joyce Maria Ferreira Alexandre Lima, Marcela Bermudez Echeverry, Carlos Alberto-Silva

**Affiliations:** 1 Natural and Humanities Sciences Center (CCNH), Experimental Morphophysiology Laboratory, Federal University of ABC (UFABC), São Bernardo do Campo 09606-070, Brazil; brendarufino33@gmail.com (B.R.d.S.); joycealexandre02@outlook.com (J.M.F.A.L.); 2 Center for Mathematics, Computation and Cognition (CMCC), Federal University of ABC (UFABC), São Bernardo do Campo 09606-070, Brazil

**Keywords:** antipsychotics, striatum, hippocampus, septo-hippocampal cholinesterase, AChE inhibition

## Abstract

Background/Objectives: Antipsychotic medicines are used to treat several psychological disorders and some symptoms caused by dementia and schizophrenia. Haloperidol (Hal) is a typical antipsychotic usually used to treat psychosis; however, its use causes motor or extrapyramidal symptoms (EPS) such as catalepsy. Hal blocks the function of presynaptic D2 receptors on cholinergic interneurons, leading to the release of acetylcholine (ACh), which is hydrolyzed by the enzyme acetylcholinesterase (AChE). Methods: This study was designed to investigate the Hal-inhibitory effects on AChE activity in regions representative of the cholinergic system of mice and potential associations between cataleptic effects generated by Hal using therapeutic doses and their inhibitory effects on AChE. Results: The distribution of the AChE activity in the different regions of the brain followed the order striatum > hippocampus > (prefrontal cortex/hypothalamus/ cerebellum) > brainstem > septo-hippocampal system. In ex vivo assays, Hal inhibited AChE activity obtained from homogenate tissue of the striatum, hippocampus, and septo-hippocampal system in a concentration-dependent manner. The inhibitory concentration of 50% of enzyme activity (IC_50_) indicated that the septo-hippocampal system required a higher concentration of Hal (IC_50_ = 202.5 µmol·L^−1^) to inhibit AChE activity compared to the striatum (IC_50_ = 162.5 µmol·L^−1^) and hippocampus (IC_50_ = 145 µmol·L^−1^). In in vivo assays, male Swiss mice treated with concentrations of Hal higher than 0.1 mg·kg^−1^ induced cataleptic effects. Positive correlations with *Spearman’s correlation* were observed only between the lack of cataleptic effect and the decreased AChE activity of the hippocampus in the mice treated with 0.01 mg·kg^−1^ of Hal but not in the striatum and septo-hippocampal system. Conclusions: Our results suggest that Hal could increase cholinergic effects via AChE inhibition, in addition to its dopamine antagonist effect, as an alternative approach to the treatment of behavioral disturbances associated with dementia.

## 1. Introduction

Antipsychotic medicines are used to treat a variety of psychological disorders, including schizophrenia [1,2]. They are also used to treat some dementia-related symptoms, such as those seen in Alzheimer’s disease (AD), especially psychosis, agitation, and violence [1,2]. There are two categories of antipsychotics, typical and atypical, which differ in their action and side effects [2]. The first antipsychotic drugs, commonly referred to as typical antipsychotics (such as chlorpromazine and haloperidol), were primarily characterized by their ability to antagonize dopaminergic D2 receptors (D2R). This pharmacological action is associated with a range of adverse motor implications [3,4]. Atypical antipsychotics (clozapine and olanzapine, for example) were developed a few years later to try to reduce the deleterious motor effects found with typical antipsychotics; they have a stronger interaction with receptors, including activity on serotonin, dopaminergic, and adrenergic receptors [4,5].

Haloperidol (Hal) is a typical antipsychotic drug employed in the treatment of many mental health conditions [6] due to its effectiveness, relatively lesser sedative and hypotensive effects, and fewer anticholinergic properties [7,8]. However, it is associated with the occurrence of unattractive motor or extrapyramidal symptoms (EPSs) such as akinesia, catalepsy, bradykinesia, and muscular stiffness [9,10]. Studies have reported that motor deficits are likely a result of the antagonistic effects of Hal on the striatal dopaminergic system [10,11]. Antipsychotics induce cataleptic effects by blocking postsynaptic D2R in the indirect striatal pathway and presynaptic D2R on cholinergic interneurons, resulting in an increase in acetylcholine (ACh) signaling on striatal projection neurons [12]. Therefore, the presence of ACh in the synaptic cleft initiates the activation of the cholinergic synapse, which is hydrolyzed by the enzyme acetylcholinesterase (AChE). This process results in the release of acetate and choline, which are then reabsorbed by the presynaptic terminal to facilitate the synthesis of further ACh [2,13,14]. AChE is distributed throughout the central nervous system. The principal regions with high AChE activity include the prefrontal cortex, hippocampus, and striatum [14,15].

AChE inhibitors (AChEIs) prevent the hydrolysis of ACh, resulting in a reduction in the activity of AChE [14]. Schizophrenia and some dementias course with visual hallucinations that may arise from a dysregulation of the brain’s cholinergic system (nicotinic and muscarinic), being treated with the AChEIs, with effects about the same psychosis [16]. The possible inhibitory effects of 26 clinically available antipsychotics, including Hal, on the activity of recombinant human AChE were described in the literature to predict the function of antipsychotic-induced AChE inhibition in the development of EPSs [17]. Although Hal has been found to have anticholinergic effects [18,19,20,21], it is important to note that AChE inhibition only occurs at high concentrations. This implies that if the appropriate dosage is followed, it is rare for Hal to elicit EPSs associated with AChE inhibition [17]. The use of Hal as a therapeutic intervention for schizophrenia has been widely practiced for over six decades [16]. However, there is still little information regarding its influence on AChE, specifically in terms of its inhibitory potential. That said, the objective of this study was to assess the effects of the antipsychotic drug Hal on the AChE activity of a commercial enzyme from *Electrophorus electricus* or on the total homogenate protein of different regions of the brain in mice associated with typical antipsychotic-induced EPS. Additionally, the study also involved the administration of three therapeutic doses to mice, with one of the doses specifically intended to induce cataleptic symptoms in order to investigate potential associations between cataleptic effects generated by Hal and inhibitory effects on AChE.

## 2. Materials and Methods

### 2.1. Reagents and Solutions

Acetylthiocholine iodide, Acetylcholinesterase, Tris (hydroxymethyl)-aminomethane GR, 5,5-dithiobisnitrobenzoic acid (DTNB), and Coomassie brilliant blue G were obtained from Sigma Chemical Co (St. Louis, MO, USA) and bovine serum albumin and K_2_HPO_4_ from Merck-Millipore, Darmstadt, Germany. The Hal antipsychotic (Haldol^®^) was purchased from Janssen-Cilag (Beerse, Belgium). The remaining chemical reagents utilized in the current investigation were of analytical quality, with a purity above 95%, and were obtained from reputable commercial suppliers.

### 2.2. Animals and Drug

We acquired male Swiss mice, aged 7 to 8 weeks, with a body weight ranging from 30 to 35 g, from our institution. The mice were housed in groups of 3–4 per cage with environmental enrichment and underwent a quarantine period of 7 days. The animals were exposed to a 12 h light/dark cycle and continual exhaust ventilation using the Alesco^®^ system from Brazil, with temperature and humidity-controlled. They were provided with standardized mouse chow (Nuvital Nutrientes Ltd., Curitiba, Brazil) and had unlimited access to it. Haloperidol was administered acutely via intraperitoneal injection 1, 0.1, and 0.01 mg·kg^−1^ (Haldol^®^ drops solution) or saline as a vehicle control group. The experimental protocols were performed in accordance with the guidelines for the use of laboratory animals in Biochemical Research and were approved by the Ethics Committee on Animal Use (CEUA—UFABC) protocol number 2511041218. All efforts were made to minimize animal suffering.

### 2.3. Effects of Hal on Commercial Enzyme AChE

The AChE enzymatic activity was determined by a modification of Ellman’s method [22]. In a 96-well plate (Nest Biotechnology, Rahway, NJ, USA), Hal was added at different concentrations (250 to 4 µmol·L^−1^), AChE (1 U·mL^−1^) (Sigma, St. Louis, MO, USA), and 160 μL of 5,5-dithiobis-2-nitrobenzoic acid (0.33 mmol·L^−1^; DTNB, Ellman’s reagent; Sigma, St. Louis, MO, USA) in phosphate buffer (0.1 mol·L^−1^, pH 8.0) to a final volume of 190 μL per well. The experiments were performed in triplicate and incubated at room temperature for 10 min. Next, we added 10 μL of acetyltiocholine iodide (20 mmol·L^−1^) (Sigma, St. Louis, MO, USA). The hydrolysis of acetylthiocholine iodide was monitored at 412 nm for 20 min in a microplate reader (BioTek Instruments, Inc., Winooski, VT, USA). The AChE activity was expressed as the concentration of acetylthiocholine iodide substrate hydrolyzed in µmol·min·mg^−1^. Data were presented as mean ± SEM of two independent experiments in triplicate.

### 2.4. AChE Activity Distribution in Different Tissues of the Brain and Inhibitory Effects of Hal

The animals (n = 6) were euthanized by cervical dislocation performed by a trained veterinarian to avoid anesthesia use and interference. Tissue brains were quickly removed and placed on ice. The striatum, hippocampus, septo-hippocampal system, brainstem, prefrontal cortex, hypothalamus, and cerebellum were gently dissected and immediately frozen in liquid nitrogen and stored at −80 °C until tissue processing. Briefly, the brain regions were homogenized in a glass potter in a solution of 10 mmol·L^−1^ Tris–HCl, pH 7.4, on ice, at a proportion of 1:50 (*w*·*v*^−1^), according to Pimentel et al. [22]. The homogenate was centrifuged at 3000× *g* for 10 min, and the resulting supernatant was utilized. Protein was determined previously by the Bradford method [23] and adjusted for all tissues (0.8 mg·mL^−1^). The AChE enzymatic assay in the different regions of the brain was determined by Ellman’s method [24] and described below. In addition, the ex vivo effects of Hal on the striatum, hippocampus, and septo-hippocampal system were also analyzed. Briefly, different concentrations of Hal 250 to 4 µmol·L^−1^, homogenate of different tissue (0.8 mg·mL^−1^), DTNB 0.33 mmol·L^−1^ (Ellman’s reagent; Sigma, St. Louis, MO, USA) were added in triplicate in a 96-well plate (Nest Biotechnology, Rahway, NJ, USA) in a volume of 200 µL. Samples were incubated at room temperature for 10 min, and after adding acetyltiocholine iodide (20 mmol·L^−1^). The hydrolysis of acetylthiocholine iodide was monitored at 412 nm for 20 min in a microplate reader (BioTek Instruments, Inc., Winooski, VT, USA). The AChE activity was expressed as the concentration of acetylthiocholine iodide substrate hydrolyzed in different brain areas in µmol·min·mg^−1^. We calculated the percentage of AChE inhibition (I) using the formula [(absorbance for the control—absorbance for the sample)/absorbance for the control] × 100. Additionally, we also determined the IC_50_ (concentration that inhibits 50% of AChE activity) by performing linear regression using the mean of three assays.

### 2.5. Catalepsy Test and AChE Activity in Striatum, Hippocampus, Septo-Hippocampal System

Albino male mice (Swiss) were assigned to groups (4–5 animals per group) and treated with Hal (0.01, 0.1, or 1 mg·kg^−1^) diluted in sterile 0.5% NaCl (*w*·*v*^−1^). Hal samples were injected intraperitoneally (i.p.). The control group received only sterile 0.5% NaCl (*w*·*v*^−1^, 10 mL·kg^−1^) under the same conditions reported earlier. The doses of Hal used in the experiments were in agreement with those reported in the literature [5,25,26]. The catalepsy method, which consists of placing both of the animal’s forelimbs over a horizontal cylindrical glass bar (diameter: 0.5 cm; height: 4.5 cm above the table), was examined 30, 60, and 120 min after administration [5,27]. The amount of time (in seconds—s) that both forelimbs remained on the bar was measured up to 300 s [28]. Catalepsy was regarded as having ended when the mouse’s forepaws hit the floor or climbed up the bar. After 120 min of treatment, the animals were euthanized, as described above, and the striatum, hippocampus, and septo-hippocampal system were dissected using a stereo loupe with LED light illuminator and immediately stored at −80 °C until tissue processing. Then, the homogenates of tissues were obtained according to Pimentel and collaborators [22], and AChE enzymatically determined by Ellman’s method [24], as described below. Data were shown as mean ± SEM or box-and-whisker plots of experimental groups assayed in triplicate for each animal.

### 2.6. Statistical Analyses

The statistical analysis was carried out using the GraphPad Prism 6.0 software (GraphPad Software, Inc., La Jolla, CA, USA) or JASP software (JASP Team, Version 0.16.2, Amsterdam, The Netherlands). Data were analyzed using repeated measures ANOVA for behavior and one-way analysis of variance (ANOVA) for between-group comparisons, followed by Tukey’s post hoc test for multiple comparisons. Spearman’s correlation coefficient (r, range of values +1 to −1) was utilized to investigate the relationship between catalepsy tests and AChE activity in the striatum, hippocampus, and septo-hippocampal system tissues. After determining the correlation coefficient, linear regression was used to determine the relevance of each predictor. Data from catalepsy tests over 120 min in the different experimental groups were used for correlations. Values of *p* < 0.05 were considered statistically significant in all analyses. Jeffrey’s Amazing Statistics Program (JASP) was used to generate a correlation heatmap (Spearman’s r correlation matrix) based on pairwise correlations.

## 3. Results

### 3.1. Inhibitory Effects of Hal on the Commercial Enzyme AChE from Electrophorus electricus

**Figure 1:**
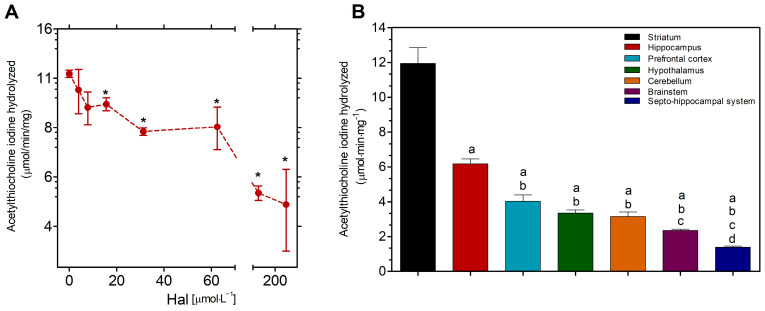
The impact of Hal on the commercial enzyme AChE from *Electrophorus electricus* (**A**) and the distribution of AChE activity in several mouse brain regions (**B**). The AChE activity was expressed as mean ± SEM from six independent animals per group. Data were analyzed by one-way statistical ANOVA followed by Tukey’s post-test. *p* < 0.05 was considered significant and represented as “*” in relation to the absence of Hal, “a” in relation to the striatum, “b” in relation to the hippocampus, “c” in relation to the prefrontal cortex, and “d” in relation to the hypothalamus.

Hal inhibited commercial AChE activity in a dose-dependent manner, with a significant difference (*p* < 0.05) from 15.8 to 200 µmol·L^−1^ in comparison to the control (Figure 1A).

### 3.2. Distribution of AChE Activity in Several Mouse Brain Regions

The striatum showed the highest levels of AChE activity (11.8 ± 3.2 µmol·mg^−1^), followed by the hippocampus (6.1 ± 0.9 µmol·mg^−1^) in relation to the others (Figure 1B). The distribution of the AChE activity in the different regions of the brain followed the order striatum > hippocampus > (prefrontal cortex/hypothalamus/cerebellum) > brainstem > septo-hippocampal system when the data were examined using one-way ANOVA followed by Tukey’s post hoc test for multiple comparisons (*p* < 0.05).

### 3.3. Inhibitory Effects of Hal on AChE Activity In Vitro in Homogenate Obtained from Striatum, Hippocampus, and Septo-Hippocampal System

**Figure 2:**
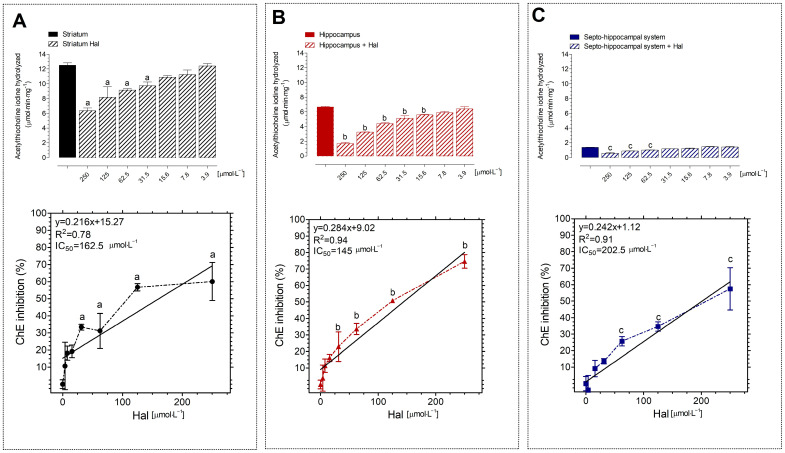
Effects of Hal on AchE activity in homogenate obtained from striatum (**A**), hippocampus (**B**), and septo-hippocampal system (**C**). Hal reduced AChE activity in three tissues in dose- and concentration-dependent manners. Data were analyzed by one-way statistical ANOVA followed by Tukey’s post-test; A significance level of *p* < 0.05 was considered statistically significant and represented by “a” in relation to AChE activity in the striatum, “b” in relation to AChE activity in the hippocampus, and “c” in relation to AChE activity in the septo-hippocampal system.

The effects of Hal at various concentrations on AChE activity were studied in homogenates of corpus striatum, hippocampus, and septo-hippocampal system regions (Figure 2). Hal decreased the enzyme activity in a dose-dependent effect in all tissues tested. When compared to the control group, AChE inhibitory effects occurred at concentrations more than 31.5 µmol·L^−1^ in the striatum, and the IC_50_ was determined at 162.5 µmol·L^−1^ (Figure 2A).

AChE inhibition was seen at concentrations more than 15.5 µmol·L^−1^ of Hal, with the IC_50_ in the hippocampus being 145 µmol·L^−1^ (Figure 2B). The IC_50_ in the septo-hippocampal system was 202.5 µmol·L^−1^, and AChE inhibition was seen at doses greater than 62.5 µmol·L^−1^ of Hal when compared to the control group (Figure 2C). The IC_50_ values demonstrated that the septo-hippocampal system requires a greater concentration of Hal (IC_50_ = 202.5 µmol·L^−1^) to inhibit AChE activity, followed by the striatum (IC_50_ = 162.5 µmol·L^−1^) and hippocampus (IC_50_ = 145 µmol·L^−1^).

### 3.4. Effects of Hal on Catalepsy

**Figure 3:**
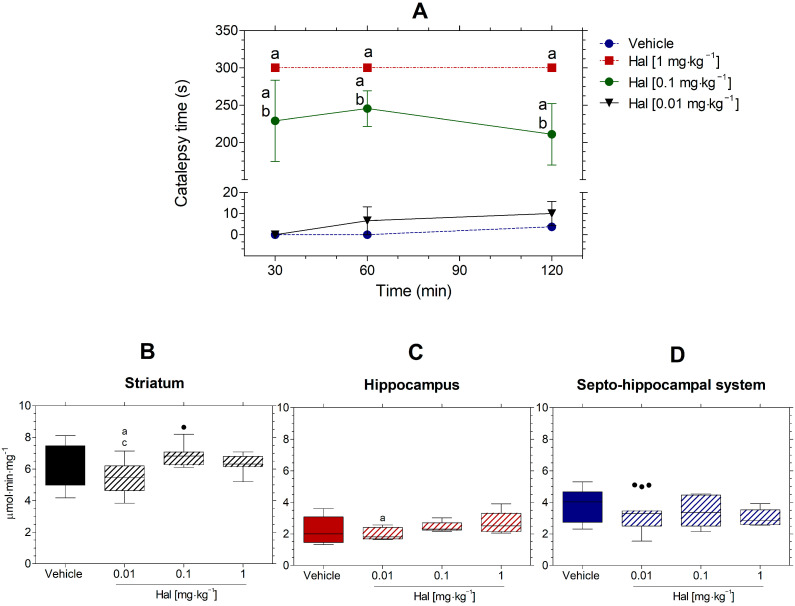
Cataleptic effects of Hal (**A**) and AChE activity after 120 min of treatment in the form striatum (**B**), hippocampus (**C**), and septo-hippocampal system (**D**). Hal induced cataleptic effect when compared with the control group, except with the 0.01 mg·kg^−1^. Cataleptic effects of Hal were presented as means ± SEM, and the AChE activities in the striatum, hippocampus, and septo-hippocampal system as box-and-whisker plots. Statistical analysis was performed with two-way repeated-measures ANOVA and Bonferroni post hoc test (N = 4–5 per group). A significance level of *p* < 0.05 was considered statistically significant and represented by “a” in relation to the vehicle, “b” in relation to Hal 1 mg·kg^−1^, and “c” in relation to Hal 0.1 mg·kg^−1^. Points represent the means ± SEM.

Hal was administered to the mice in doses of 1, 0.1, and 0.01 mg·kg^−1^, and catalepsy was observed at intervals of 30, 60, and 120 min (Figure 3A). In comparison to the vehicle group, Hal at 1 mg·kg^−1^ produced significant catalepsy from 30 to 120 min after the administration, with 300 seg of catalepsy recorded. At 0.1 mg·kg^−1^, Hal also had catalepsy from 30 min to 120 min, but it was lower than the group treated with 1 mg·kg^−1^. Mice treated with Hal at 0.01 mg·kg^−1^ did not exhibit catalepsy at any point of evaluation [RM ANOVA, Factor time, F (2,28) = 3.445; *p* = 0.046; treatment variable, F (3,14) = 103.466; *p* < 0.001; Tuckey test, *p* < 0.05] (Figure 3A).

### 3.5. Effects of Hal on AChE Activity In Vivo in Striatum, Hippocampus, and Septo-Hippocampal System

Mice treated with vehicle, 1, 0.1, or 0.01 mg·kg^−1^ of Hal were euthanized at 120 min, as mentioned before, and a measure of AChE activity in the striatum, hippocampus, and septo-hippocampal system was carried out (Figure 3B–D). Mice treated with Hal at 0.01 mg·kg^−1^ decreased the AChE activity in the striatum compared to the vehicle group and 0.1 mg·kg^−1^ of Hal (Figure 3B). In the hippocampus, AChE activity was also decreased when mice were treated with 0.01 mg·kg^−1^ in relation to the vehicle (Figure 3C). In contrast to the striatum and hippocampus, no changes in AChE activity were detected in all doses tested in the septo-hippocampal system (Figure 3D).

### 3.6. Correlations Analyses

**Figure 4:**
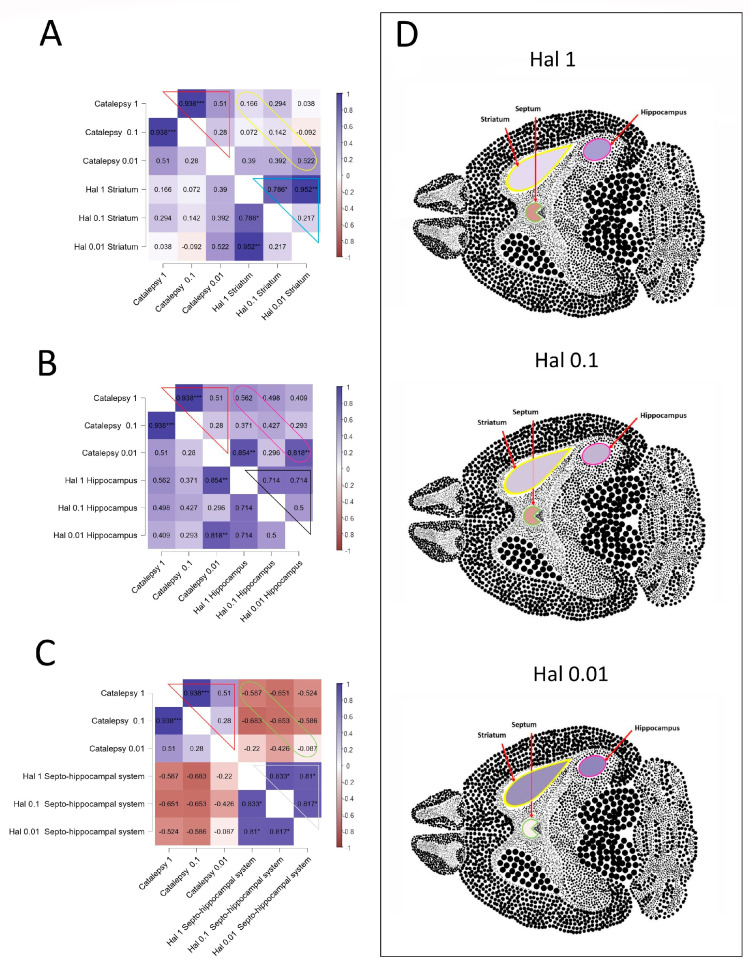
Spearman correlation analyses between catalepsy and AChE activity in the striatum (**A**), hippocampus (**B**), and septo-hippocampal system (**C**). The positively correlated variables are represented by the blue tone, and the negatively correlated variables are represented by the wine tone (Spearman rho from +1 to −1). The correlations between catalepsy and AChE activity in the striatum, hippocampus, and septo-hippocampal system (yellow, pink, and green lines, respectively) or between different doses of Hal in the catalepsy assay (red line) or between different doses of Hal in the AChE activity in the striatum, hippocampus, and septo-hippocampal system (blue, black, and white lines, respectively) were indicated in the different color lines. (**D**) Schematic representative correlations at each dose were shown in the striatum, hippocampus, and septo-hippocampal system sections. JASP software for correlations was employed to perform Spearman’s analyses, and significant differences were set at * *p* < 0.05, ** *p* < 0.01, *** *p* < 0.001.

The Spearman correlations between catalepsy and AChE activity in the striatum, hippocampus, and septo-hippocampal system induced by Hal are represented in Figure 4, and complete analyses of correlations are in the Supplementary Material (File S1). The concentration-dependent effects of Hal on catalepsy were seen while evaluating the correlation between doses tested (Figure 4A–C; red line). In the striatum, a strong positive correlation was identified between the AChE activity in the mice treated with 1 and 0.1 mg·kg^−1^ of Hal (Rho = 0.786; *p* = 0.028) and between the doses 1 and 0.01 mg·kg^−1^ of Hal (Rho = 0.952; *p* = 0.001) (Figure 4A; blue line). However, there were no significant alterations in the correlation between the catalepsy and AChE activity in the non-dosage of treatment with Hal (Figure 4A; yellow line). In the region of the hippocampus (Figure 4B), Hal induced a positive correlation between the lack of cataleptic effect and the decreased AChE activity in the mice treated with 0.01 mg·kg^−1^ of Hal (Rho = 0.818; *p* = 0.007) (Figure 4B; pink line), but no correlations were observed between the AChE activity and different concentrations of Hal (Figure 4B; black line). No significant correlations occurred between catalepsy and AChE activity in the septo-hippocampal system when employing the doses of Hal, as depicted in Figure 4C (green line). Nevertheless, there is a negative correlation tendency between the increase in catalepsy and the decrease in AChE activity at 1 and 0.1 mg·kg^−1^ of Hal (yellow line; Rho = −0.587, *p* = 0.126; Rho = −0.653, *p* = 0.057, respectively). Additionally, significant differences were observed between the groups of mice treated with 1 and 0.1 mg·kg^−1^ of Hal (Rho = 0.833; *p* = 0.015), between the groups treated with 1 and 0.01 mg·kg^−1^ of Hal (Rho = 0.810; *p* = 0.022), and between the groups treated with 0.1 and 0.01 mg·kg^−1^ of Hal (Rho = 0.817; *p* = 0.011) (Figure 4C; white line).

The correlations between catalepsy effects and AChE activity in the striatum, hippocampus, and septo-hippocampal system were also evaluated for each concentration separately and represented in colors in sections of the brain (Figure 4D). The correlations between the catalepsy effects and AChE activity in the three tissues showed distinct tendencies in relation to the concentration of Hal studied (Figure 4D). Reduction in positive correlations between catalepsy and AChE activity in the striatum and hippocampus was observed with the increase in Hal concentrations (Figure 4D). In contrast, increase in negative correlations between catalepsy and AChE activity in the septo-hippocampal system when Hal concentrations are increased (Figure 4D).

## 4. Discussion

Catalepsy is often considered a typical symptom of EPS effects associated with antipsychotic drugs [7,8,29]. These effects have been suggested to occur due to the inhibitory impact of these medications on indirect pathways or striatopallidal neurons, which predominantly express D2Rs. Additionally, there may be a direct enhancement of cholinergic actions when Hal blocks the presynaptic inhibitory D2R signaling on cholinergic interneurons, such as through the inhibition of AChE [30,31,32]. For the first time, we described the inhibitory effects of Hal on AChE activity in the striatum, hippocampus, and septo-hippocampal system, brain-specific regions with significant cholinergic system activity, and it is known that their dysregulation is involved in the mechanism of catalepsy [33,34], and Alzheimer’s disease [35].

AChE enzyme, which is found in cholinergic synapses with functional requirements within the synaptic cleft, regulates the development, differentiation, and plasticity of developing central nervous system neurons [36]. The distribution of the AChE activity in the different regions of the brain in mice was confirmed in our study and followed the order striatum > hippocampus > (prefrontal cortex/hypothalamus/ cerebellum) > brainstem > septo-hippocampal system. In our study, we demonstrated that Hal inhibited commercial AChE activity in a dose-dependent manner, according to a previous study reported in the literature [17]. Furthermore, Hal has also shown the ability to reduce the AChE activity in the total protein homogenate of the striatum, hippocampus, and septo-hippocampal system tissues in a dose-dependent manner. Our results suggested that there are differences in the sensitivity of the AChE inhibitory effects of Hal among the three tissues examined. The hippocampus, with an IC_50_ value of 145 µmol·L^−1^, has a higher susceptibility to the inhibitory effects of Hal on AChE compared to the septo-hippocampal system, which has an IC_50_ value of 202.5 µmol·L^−1^, for instance.

In mice treated with Hal, AChE activity was enhanced in the striatum, hippocampus, and prefrontal cortex, with minor activity in the septo-hippocampal pathway, which is involved in spatial memory impairment [37]. Cholinergic activity in the hippocampus correlates with memory, and there is a great interest in the potential of cholinergic drugs for treating impairments in memory resulting from disruption of septo-hippocampal function. Dopaminergic transmission plays a crucial role in regulating cholinergic activity in the striatum, which is essential for motor and learning processes. [38]. Studies in vivo have shown that Hal can cause a dopaminergic-cholinergic imbalance in the striatum since it has been shown that ACh levels can be reduced after acute [39] or chronic treatment [34]. Then, an increased turnover is proposed.

Like our work, a study in vitro of comparative concentrations of Hal and atypical antipsychotics such as sulpiride and olanzapine showed the ability to promote a decrease in AChE activity or inhibition. Here, also, the study in vivo with the zebrafish brain observed significantly increased activity after Hal administration [40]. Previous behavioral experiments have demonstrated that the presence of cholinergic pathways is necessary for all manifestations of Hal-induced catalepsy [41,42]. 

It is possible that Hal blocking the D2 dopamine receptors can consequently exacerbate these imbalances and induce catalepsy [42]. For these reasons, the comparison of AChE inhibitors and antipsychotics in experimental schizophrenia models has been the center of attention [43].

Nonetheless, as far as our knowledge goes, studies involving dose–response and AChE inhibiting activity in Hal-induced catalepsy were not found, nor either AChE activity in specific brain structures. In our work, it was observed that the no-cataleptic dose decreased AChE activity in the striatum and hippocampus but not in the septum. This decreased AChE activity was confirmed by a positive correlation in the hippocampus. However, in the striatum and septum, positive correlations were also observed among all doses. It is suggested that a dose that is significantly lower (Hal 0.01 mg·kg^−1^, human equivalent 0.057 mg for 70 k bw) than those used for the treatment of psychosis or the control of agitation (0.5 to 5 mg/2 or 3 times a day) (Hal 0.1 mg·kg^−1^, human equivalent 0.57 mg, and Hal 1 mg·kg^−1^, human equivalent 5.7 mg for 70 k bw, see calculation in Nair and Jacob [44]) could increase ACh in the synaptic cleft and modulate the cholinergic system in the hippocampus. This could potentially help improve cognitive impairments associated with dementia or schizophrenia without causing catalepsy. As mentioned earlier, the blockade of presynaptic D2R on cholinergic interneurons by Hal can increase ACh signaling on striatal projection neurons (MSNs—medium spiny neurons), resulting in selective ACh stimulation of M1 muscarinic receptors in the indirect pathway on D2R-MSNs. The simultaneous blockade of D2R by Hal and M1R stimulation could explain the presence of catalepsy [12]. However, our work has limitations because a single dose was administered, and subchronic or chronic effects could be explored.

## 5. Conclusions

In summary, antipsychotic drugs are prescribed to elderly patients for the treatment of various psychopathological conditions, including psychosis and agitation associated with dementia. Our results with Hal suggest that the low dose devoid of EPS (0.01 mg·kg^−1^, or its equivalent 0.05 mg in humans), as examined by the catalepsy test, decreases AChE activity in the striatum and hippocampus but not in the septum. Increased cholinergic activity in both regions is required for motor learning and spatial memory, respectively, two conditions almost absent in dementias.

## Data Availability

Data and materials are available upon request.

## Supplementary Materials

The following supporting information can be downloaded at https://www.mdpi.com/article/10.3390/neurolint16060125/s1.

## Abbreviations

ANOVA: One-way analysis of variance; EPSs: extrapyramidal symptoms; D2R: D2 dopaminergic receptor; AD: Alzheimer’s disease; AChE: enzyme acetylcholinesterase; AChEIs: AChE inhibitors; IAChE: inhibition of AChE; MSNs: *Medium* spiny *neurons*.
